# A Case of Glanders in the Human Subject

**Published:** 1852-08

**Authors:** Josiah T. Watts

**Affiliations:** Enterprise, Miss.


ART. III.—*A Case of Glanders in the Human Subject.* By JOSIAH T. WATTS, M. D., of Enterprise, Miss.

Axum Permenter, a young man about twenty-seven years of age, stout and healthy, of rather intemperate habits, had been for some time a teamster on a railroad, sleeping on the ground at night. Had a horse to die of the glanders, and in about ten days afterwards, began to complai of an unusual pain or fullness in the head, general lassitude and weakness of the whole system. Continued in this condition for three days without applying for medical aid. I saw him on the 23d of June, he complained of pain in his bowels, and had a thin bloody mucous discharge from the bowels. Believing it to be diarrhea, (for a good many of the family were suffering with this complaint at that time,) I gave him calomel and opium, conbined with kino. The diarrhea ceased under this course of treatment, and he began to improve for two days. He then complained of pain just over the right eye, with a headache; pulse full and bounding, tongue coated with a white fur; tip red, great difficulty in projecting it, the whole nervous system in a complete tremor. Bled him copiously from the right arm, applied a blister to the back of the neck, which discharged freely up to the time of his death. Ordered 10 grs. calomel, with 2 of ipecac at bed time; at 1 o'clock at night, gave ¼ gr. sulphate of morphine, under which he rested well.

24th. Next morning bowels opened twice; no fever, slight head ache, right eye swollen, and also the right nostril. Ordered during the day, quinine, nitre, and paregoric combined; skin became moist after the second dose; at night a Dover's powder. Patient rested pretty well.

25th. Pulse soft, tongue unchanged, bowels moved once; the right side of the face very much swollen, so much that he could not see out of the right eye; right nostril closed; a slight bloody yellow water issued from the eye and nose. Applied cloths wrung out of cold water medicated with acetas plumbi; gave morphine and calomel.

26th. Patient rested badly; face continues to swell; the left side swollen as much as the right; cannot see at all; gets his breath through his mouth alone; a yellow discharge mixed with blood, from both his eyes and nostrils, removes the cuticle wherever it touches; has to be propped up in bed on account of the discharge from his nostrils, which is swallowed when he is in a recumbent posture; the nose dark colored, with small ulcers over it; difficulty of swallowing; in attempting to drink water about half runs back through the nostrils; the discharge is exceedingly offensive, and similar to that of gl ndered horses. Saw him again in the evening—condition as in the morning, except that his throat is much swollen; he can scarcely utter a single word, owing to the swelling of his lips; the discharge still continues from both his eyes and nostrils as before, but is not now sanguineous. Complains only of a difficulty in breathing; the discharge is extremely offensive; nose almost black; neck swelling very fast.

27th. Died at 8 o'clock, A. M., the discharge continuing from his nose and eyes until he died.

No post-mortem examination was made. Several persons present at the time of his death remarked, that the odor of the discharge was the same as that which they had often perceived about horses with glanders.

June 30, 1852.

